# Evaluating outcomes of hyoid suspension combined with other upper airway surgery for obstructive sleep apnea

**DOI:** 10.1007/s11325-025-03370-8

**Published:** 2025-07-07

**Authors:** Christian Schaaff, Tejal H. Patel, Dania Alazawi, Evan Thomas, Mary H. Kress, Nicholas R. Lenze, Jeffrey J. Stanley, Paul T. Hoff

**Affiliations:** 1 University of Michigan Medical School, Ann Arbor, MI USA; 2 Department of Otolaryngology– Head and Neck Surgery, University of Michigan Medical School, Ann Arbor, MI USA; 3 University of Michigan Medical School, 1500 East Medical Center Drive, Ann Arbor, MI 48108 USA

**Keywords:** Hyoid bone surgery, Palate surgery, Obstructive sleep apnea, Treatment outcomes, Polysomnography

## Abstract

**Objective:**

To assess the effectiveness of hyoid suspension in adults with OSA.

**Methods:**

This is a retrospective case series of adult patients with OSA who underwent hyoid suspension at a single tertiary center. Patients were excluded if they did not have pre- and postoperative sleep study data. Change in AHI and ESS scores from preop to postop were estimated using paired t-tests. Data were analyzed using stratified analyses, simple linear regression models, and univariate logistic regression models.

**Results:**

43 patients underwent hyoid suspension from 2012 to 2023 with a mean (SD) age of 47.7 (9.5) years, mean (SD) BMI of 31.4 (6.9) kg/m2, and mean preoperative AHI of 30.6 (21.1) events/hr. Most patients had an adjuvant procedure performed concurrently, including palatal surgery (*n* = 11), genioglossus advancement (*n* = 3), or palatal surgery and genioglossus advancement (*n* = 24). There was a statistically significant improvement in the pre- and post-surgery AHI (mean decrease of -9.4 events/hr (95% CI − 17.1 to − 1.6, *p* = 0.019)) and ESS score (mean decrease of − 2.0 points (95% CI − 3.7 to − 0.3, *p* = 0.025)). Thirteen (30.2%) patients achieved success based on Sher20 criteria.

**Conclusions:**

Hyoid suspension with adjuvant OSA surgery results in statistically significant improvement in AHI and ESS scores, but additional research is needed to evaluate whether these results are clinically impactful.

**Level of Evidence:**

3.

**Supplementary Information:**

The online version contains supplementary material available at 10.1007/s11325-025-03370-8.

## Introduction

Obstructive sleep apnea (OSA) affects approximately 25% of adults in the United States and is linked with a range of health issues including cardiovascular disease, metabolic syndrome, depression, and cognitive impairment [1–6]. Current first-line treatments typically include lifestyle modifications, such as weight loss and sleep positioning, as well as positive airway pressure (PAP) [7]. However, lifestyle changes often take considerable time to alleviate symptom burden and may not fully address underlying causes of airway collapse. Furthermore, studies have shown a 25% PAP non-adherence rate, indicating that this treatment may not be helpful for a large proportion of individuals with OSA [8]. Patients can have PAP intolerance for various reasons, including nasal obstruction, lack of perceived treatment effect, feelings of claustrophobia, noise, and air leakage [9]. Given the considerable burden of OSA in the United States and the barriers to conservative treatment options, surgical treatment of OSA has an important role [10].

**Fig. 1:**
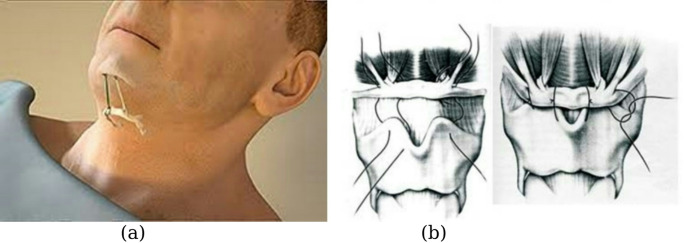
(**a**) hyoid-mandibulopexy procedure [11], (**b**) hyoid myotomy with suspension [12]

Hyoid suspension, either via the hyoid-mandibulopexy procedure, which makes use of the Airlift device (copyright by Siesta Medical Inc., Los Gatos, CA), or hyothyroidopexy procedure (see Fig. 1), is a surgical treatment option for OSA that may confer unique advantages for select patients [11, 12]. It works by surgically suspending the hyoid bone to either the thyroid cartilage or mandible, which helps to address airway collapse by stiffening the lateral pharyngeal wall, opening the hypopharynx, and anteriorly rotating the epiglottis [10]. Hyothyroidopexy reduces airway collapsibility by advancing and tethering the hyoid bone to the thyroid cartilage, whereas the hyoid-mandibulopexy procedure suspends the hyoid to the mandible [10]. In clinical practice, hyoid suspension is often combined with other surgical techniques, such as functional nasal surgery, palatal surgery, or mandibulotomy with genioglossus advancement [13].

Although hyoid suspension is commonly used as a surgical treatment option for OSA, there have been a limited number of retrospective case series evaluating its efficacy [14–19]. Even fewer studies have assessed patient factors that may predict success with hyoid suspension, or the effect of adjuvant procedures such as palatal surgery. This information would be helpful for optimizing patient selection and surgical planning. Our study aimed to build on existing literature by evaluating an institutional cohort of adult OSA patients who underwent hyoid suspension.

## Participants and methods

This study was approved by the University of Michigan Institutional Review Board (IRB#: HUM00155002). The data that support the findings of this study are available from the corresponding author upon reasonable request. Data are located in controlled access data storage at the University of Michigan.

### Patient sample

Patients at least 18 years of age with obstructive sleep apnea (OSA) who underwent hyoid suspension at the University of Michigan by two sleep surgeons (JS and PH) were identified by querying DataDirect for the CPT code 21,685. The initial search returned 76 patients. Electronic medical records were reviewed to collect information on demographics, clinical history, sleep study findings, and Epworth sleepiness scale scores. Patients without both a preoperative and postoperative sleep study were excluded (*n* = 33).

Patients with OSA were considered candidates for hyoid suspension if they were not able to tolerate continuous positive airway pressure (CPAP) devices. We defined CPAP intolerance as use less than 4 h per night for 5 nights per week or unwillingness to use the CPAP device at all, which is consistent with the Food and Drug Administration (FDA) definition used for hypoglossal nerve stimulation candidacy [20]. The decision to proceed with hyoid suspension with or without adjuvant procedures was made based on comprehensive evaluation by a sleep surgeon (JS or PH). In addition to history and physical exam, flexible laryngoscopy and drug-induced sleep endoscopy (DISE) were used to inform the decision-making process in some patients. DISE was specifically used for evaluating upper airway collapse patterns thought to be responsive to hyoid suspension (such as predominant tongue base or epiglottis collapse) or determining candidacy for alternative procedures, such as HGNS.

Other surgical options were considered, including hypoglossal nerve stimulation (HGNS). However, some patients failed to meet the body mass index (BMI) requirements for HGNS. Other patients were considered candidates for HGNS but preferred to avoid an implantable device. Decisions regarding the type of hyoid suspension procedure performed (hyothyroidopexy vs. hyoid-mandibulopexy) were made based on surgeon preference and experience over the study period.

### Variables

Neighborhood family income was estimated based on a patient’s home address in relation to US Census tracts, which was retrieved from DataDirect. Body mass index (BMI) (kg/m2) was the BMI recorded on the day of surgery. Prior sleep surgery was defined as a previous tonsillectomy, palatal procedure, tongue base or supraglottic procedure, neurostimulation procedure, or skeletal surgery specifically for OSA.

Sleep study data was abstracted from the most recent pre-operative sleep study and the most recent sleep study available after hyoid suspension. The current American Academy of Sleep Medicine scoring guidelines were used to calculate AHI based on apneas and hypopneas. Specifically, hypopneas were defined as at least a 30% reduction in airflow for 10 s and a 3% reduction in oxygen saturation or an arousal; apneas were defined as at least a 90% reduction in airflow for at least 10 s [21]. For patients undergoing home sleep studies, the respiratory event index (REI) was used as an approximation of AHI. Responder status was defined based on the Sher20 criteria (at least a 50% reduction in AHI and post-operative AHI < 20 events/hour).

Adjuvant OSA procedures were procedures done during the same operative encounter as the hyoid suspension. These included palatal procedures (tonsillectomy, uvulopalatopharyngoplasty, and expansion pharyngoplasty) and mandibulotomy with genioglossus advancement. Nasal procedures (septoplasty and inferior turbinate reduction) were not considered adjuvant OSA procedures based on prior literature showing negligible effect on sleep study parameters [21–23]. 

### Statistical analysis

A sensitivity analysis was performed to compare patients excluded (*n* = 33) due to missing sleep study data with the rest of the sample (*n* = 43). Change in AHI, oxygen nadir, and ESS scores from pre-op to post-op were estimated using paired t-tests. A stratified analysis was performed to compare mean (SD) change in AHI, oxygen nadir, and ESS scores and surgical success based on Sher20 criteria across adjuvant OSA procedures as well as hyoid suspension techniques (hyothyroidopexy vs. hyoid-mandibulopexy). Outcomes for the subset of patients who underwent nasal procedures were also analyzed.

Simple linear regression models were used to estimate the association of predictor variables with change in AHI. A negative beta-coefficient represents a favorable change in AHI (decrease from preop to postop) for every one unit increase in the predictor variable. Univariate logistic regression models were used to estimate associations between predictor variables and the odds of surgical success (based on Sher20 criteria). Thresholds for defining categorical variables were based on the median value for the sample (e.g. median age of 48). Race was excluded from the models due to sparse observations/complete concordance. For the subset of patients with available pre-operative DISE data, we summarized patterns of collapse at each site and evaluated associations with odds of surgical success according to Sher20 criteria. We performed separate univariate logistic regression analyses to estimate associations between predictor variables and the odds of surgical success (based on Sher20 criteria). Statistical significance was set at *p* < 0.05 for all analyses. SAS software version 9.4 (Cary, NC) was used for all analyses.

## Results

### Baseline characteristics

Of the 76 patients identified in our medical records, 43 adult OSA patients who underwent hyoid suspension surgery between 2012 and 2023 and had available sleep studies were included in this study. In a sensitivity analysis, there was no difference in age (*p* = 0.272), sex (*p* = 0.196), race (*p* = 0.494), or BMI (*p* = 0.443) between those excluded due to missing sleep study data (*n* = 33) and those included in the sample (*n* = 43).

Baseline characteristics are summarized in Table 1. The mean age of participants was 47.7 years old (SD 9.5); 34 (79.1%) were male and 90.7% identified as White. Most patients had employer-based insurance (67.4%), and the mean neighborhood family income was $92,360 (27,880). The mean preoperative BMI was 31.4 kg/m2 (SD 6.9), and the mean preoperative AHI was 30.6 events/hr (SD 21.1). For the pre-operative sleep studies, 36 participants (83.7%) underwent an in-lab polysomnogram and 7 participants (16.3%) underwent a home sleep test. Postoperatively, 31 patients underwent an in-lab polysomnogram (72.1%) and 12 participants (27.9%) underwent a home sleep test. Pre-operative DISE data was available for 16 patients (37.2%) and findings are summarized in Supplemental Table 1.

**Table 1:** Baseline characteristics

Age (mean, SD)	47.7 (9.5)
Sex	
Male	34 (79.1%)
Female	9 (20.9%)
Race	
Asian	1 (2.3%)
Black/African American	2 (4.7%)
White	39 (90.7%)
Other	1 (2.3%)
Insurance	
Employer-based	29 (67.4%)
Medicaid	3 (7.0%)
Medicare	9 (20.9%)
Other	2 (4.7%)
Neighborhood family income (mean, SD)	92,360 (27,880)
BMI (kg/m2) (mean, SD)	31.4 (6.9)
Friedman tongue position	
1	2 (6.7%)
2	4 (13.3%)
3	22 (73.3%)
4	2 (6.7%)
Prior sleep surgery	14 (32.6%)
Method of Hyoid Suspension	
Hyoid-Mandibulopexy	5 (11.6%)
Hyothyroidopexy	38 (88.4%)
PAP Pressure (mean, SD)	10.2 (3.6)
Preop AHI (mean, SD)	30.6 (21.1)
Preop O2 nadir (mean, SD)	82.4 (13.0)
Preop Epworth Sleepiness Scale Score (mean, SD)	9.2 (5.1)
Adjuvant surgeries*	
Hyoid suspension with adjuvant palatal surgery	11 (25.6%)
Hyoid suspension with adjuvant genioglossus advancement	3 (7.0%)
Hyoid suspension with adjuvant palatal surgery and genioglossus advancement	24 (55.8%)
Hyoid suspension alone	5 (11.6%)

The majority of participants underwent a hyothyroidopexy (88.4%) and the remainder underwent an hyoid-mandibulopexy procedure (11.6%). Additionally, the majority of the participants underwent adjuvant OSA-directed surgeries at the time of hyoid suspension, including palatal surgery (25.6%), genioglossus advancement (7.0%), or both palatal surgery and genioglossus advancement (55.8%). Of the 5 total hyoid-mandibulopexy procedures performed, 2 were performed with adjuvant palatal procedures.

The average operative time was 156.3 min (SD 57.9 min). The average time to postop sleep study was 8.6 months (SD 10.7 months), and the average time to postop ESS score was 11.4 months (SD 17.7 months). A total of 16 (37.2%) patients had an adjuvant nasal procedure at the time of hyoid suspension (septoplasty or inferior turbinate reduction), which was not mutually exclusive with other adjuvant procedures. Outcomes for patients who had adjuvant nasal procedures are reported in Supplemental Table 2.

### Objective and subjective outcomes

Among all patients in the sample, there was a mean decrease in AHI of 9.4 events/hr (95% CI -17.1 to -1.6; *p* = 0.019) and a mean decrease in ESS score of -2.0 (95% CI -3.7 to -0.3; *p* = 0.025) from preop to postop (Table 2). There was no statistically significant change in O2 nadir. Overall, 13 (30.2%) patients achieved surgical success based on Sher20 criteria. Overall, the number of patients with preexisting OSA with mild OSA disease burden as defined by AHI between 5 and 15 increased from 10 (23%) preoperatively to 24 (56%) postoperatively (Table 3).

**Table 2:** Change in AHI, O2 nadir, and ESS from pre- to Postop

	Preoperative (mean, SD)	Postoperative (mean, SD)	Mean difference (95% CI)	*p*-value
AHI	30.6 (21.1)	21.3 (23.4)	-9.4 (-17.1 to -1.6)	0.019
O2 Nadir	82.4 (13.0)	85.4 (7.1)	3.0 (-0.14 to 6.13)	0.060
ESS	9.2 (5.1)	6.7 (5.3)	-2.0 (-3.7 to -0.3)	0.025

**Table 3:** Number of subjects by pre- and postoperative OSA disease burden

OSA disease burden	Preoperative (%)	Postoperative (%)
Mild (AHI 5–15)	10 (23%)	24 (56%)
Moderate (AHI 15–30)	15 (35%)	10 (23%)
Severe (AHI > 30)	18 (42%)	9 (21%)
Total	43	

When stratified by adjuvant OSA procedure, patients who had hyoid suspension and a palatal procedure (*n* = 11) had the largest magnitude mean change in AHI (-28.6 events/hour (SD 24.8 events/hr)) (Table 3). In patients who underwent hyoid suspension alone (*n* = 5), the mean change in AHI was − 10.5 events/hr (SD 19.1) (Table 4). Outcomes for patients who underwent adjuvant nasal procedures are summarized in Supplemental Table 2. Outcomes stratified by hyoid suspension technique (hyothyroidopexy vs. hyoid-mandibulopexy) are summarized in Supplemental Table 2.

**Table 4:** Stratified analysis based on adjuvant procedure

	Overall (*n* = 43)	Palatal Procedure (*n* = 11)	Mandibulotomy with genioglossus advancement (*n* = 3)	Both palatal procedure and genioglossus advancement (*n* = 24)	Hyoid suspension alone (*n* = 5)
Preoperative AHI (mean, SD)	30.6 (21.1)	42.3 (25.3)	25.2 (11.5)	27.3 (19.0)	24.5 (20.3)
Change in AHI (mean, SD)	-9.4 (25.2)	-28.6 (24.8)	8.3 (10.9)	-2.5 (23.5)	-10.5 (19.1)
Change in O2 Nadir (mean, SD)	3.0 (9.7)	6.3 (7.4)	15.0 (25.3)	-0.45 (6.2)	4.3 (7.6)
Change in ESS (mean, SD)	-2.0 (3.4)	-2.3 (3.2)	n/a	-1.6 (3.3)	-2.3 (5.5)
Success based on Sher_20_ criteria	13 (30.2%)	5 (45.5%)	0 (0%)	7 (29.2%)	1 (20.0%)

### Predictors of change in AHI and surgical success

**Fig. 2:**
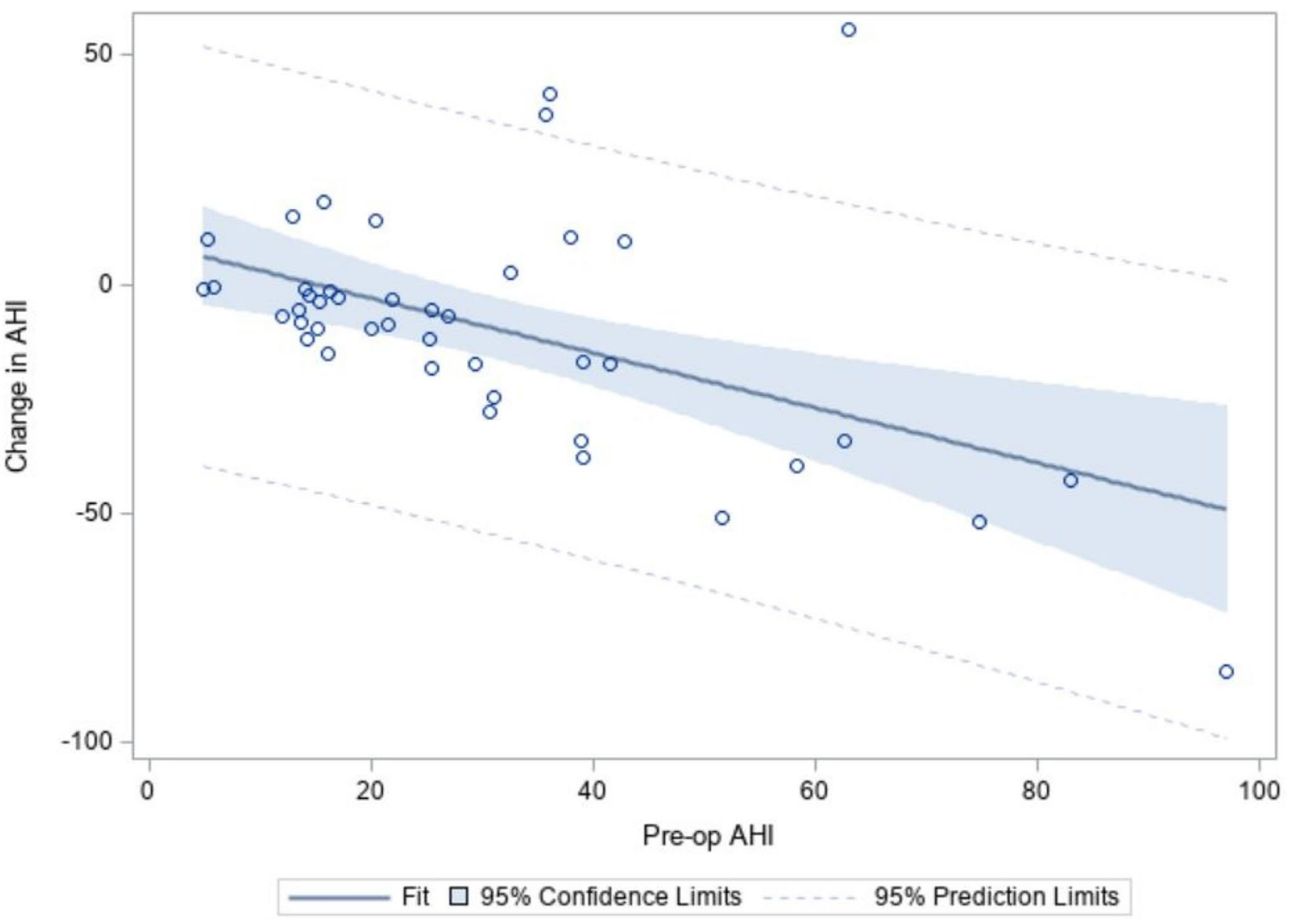
Change in AHI as a function of preoperative AHI

In simple linear regression models estimating predictors of change in AHI, higher preoperative AHI was correlated with greater reduction in AHI following surgery (β = -0.60; 95% CI: -0.92 to -0.27; *p* < 0.001) (Table 5; Fig. 2). There were no significant associations with age, sex, insurance status, neighborhood median family income, BMI, prior sleep surgery, Friedman Tongue Position, preoperative PAP pressure, surgical technique, or O2 nadir. There were no sociodemographic or clinical predictors of surgical success based on Sher20 criteria (Table 6). In patients with available pre-operative DISE data, there were no significant associations between patterns of collapse and surgical success (Supplemental Table). In patients with available pre-operative DISE data, there were no significant associations between patterns of collapse and surgical success (Supplemental Table 1).

**Table 5:** Simple linear regression models evaluating associations with change in AHI

Variable	Beta-coefficient and 95% CI	*p*-value
Age	0.14 (-0.69 to 0.98)	0.728
Female sex (vs. male)	3.77 (-15.5 to 23.0)	0.695
Insurance (vs. employer-based)		
Medicaid	2.82 (-28.1 to 33.7)	0.855
Medicare	15.8 (-3.61 to 35.2)	0.108
Other	14.8 (-22.4 to 52.0)	0.427
Neighborhood family income*	0.21 (-0.04 to 0.47)	0.098
BMI	0.95 (-0.20 to 2.10)	0.102
Friedman tongue position	1.58 (-14.1 to 17.3)	0.838
Prior sleep surgery	5.89 (-10.8 to 22.6)	0.479
Hyoid-mandibulopexy (vs. Hyothyroidopexy)	-12.1 (-36.3 to 12.1)	0.317
PAP Pressure	1.25 (-1.65 to 4.15)	0.384
Preop AHI	-0.60 (-0.92 to -0.27)	< 0.001
Preop O2 nadir	-0.10 (-0.71 to 0.52)	0.755

*per 1,000 increase

**Table 6:** Univariate logistic regression models evaluating predictors of success based on Sher_20_ criteria

Variable	Odds ratio and 95% CI	*p*-value
Age > 48 (vs. less than or equal to 48)	0.55 (0.15 to 2.06)	0.373
Female sex (vs. male)	0.60 (0.11 to 3.36)	0.559
Insurance (vs. employer-based)*		
Medicaid	0.71 (0.06 to 8.73)	0.788
Neighborhood family income >$93,760 (vs. less than or equal to $93760)	0.80 (0.21 to 3.0)	0.736
BMI > 30.6 (vs. less than or equal to 30.6)	0.51 (0.13 to 1.93)	0.320
Friedman tongue position 3–4 (vs. 1–2)	1.43 (0.22 to 9.38)	0.710
Prior sleep surgery	0.89 (0.22 to 3.61)	0.869
Hyoid-Mandibulopexy (vs. Hyothyroidopexy)	1.64 (0.24 to 11.2)	0.615
PAP Pressure > 9 (vs. less than or equal to 9)	0.71 (0.16 to 3.23)	0.662
Preop AHI > 25.5 (vs. less than or equal to 25.5)	1.53 (0.41 to 5.64)	0.527
Preop O2 nadir > 84 (vs. less than or equal to 84)	2.04 (0.51 to 8.10)	0.313

*Medicare and Other insurance omitted due to sparse observations/complete separation

## Discussion

Our study of 43 adult OSA patients who underwent hyoid suspension is one of the largest case series to date. Primary findings included a significant reduction in both AHI and ESS score following hyoid suspension, and an overall surgical success rate of approximately 30% based on Sher20 criteria. Notably, patients who underwent hyoid suspension and adjuvant palatal surgery (e.g. tonsillectomy, uvulopalatopharyngoplasty, and expansion pharyngoplasty) had the largest magnitude change in AHI. Patients with higher preoperative AHI had the greatest reduction in AHI following surgery. Overall, these findings illustrate that hyoid suspension, particularly with adjuvant palatal surgery, may be a viable surgical option for select OSA patients.

Although the change in AHI and ESS score was statistically significant in our cohort, the improvements were more modest compared to other studies [14–16, 18, 19]. Our sample had a relatively lower mean preoperative AHI (30.2 events/hour) compared to other studies, which could partially explain these differences [14–16, 18, 19]. This is supported by a study by Shaikh et al. which found a similar mean reduction in AHI (7.8 events/hr) with hyoid suspension; they reported a similar preoperative AHI to our sample (median 33.0 events/hr), and they also observed that larger preoperative AHI was associated with greater reduction in AHI [14]. Other potential contributors to the differences in outcomes between studies could be differences in baseline BMI, types of adjuvant procedures performed, or surgical technique.

Findings from our stratified analysis suggest that patients who had adjuvant palatal surgery achieved the best outcomes, with a mean reduction in AHI of − 28.6 events/hr and surgical success rate of 45.5%. These findings mirror those reported by Tantawy et al., who found a large reduction in AHI (68.4 events/hr to 25.6 events/hr) after hyoid suspension with tonsillectomy and “palatal suspension.” [16] Panah et al. observed a more modest decrease in AHI among patients receiving hyoid suspension paired with UPPP (20.2 events/hr to 10.0 events/hr), although this may partially be explained by a lower baseline AHI [17]. Of note, patients in our adjuvant palatal procedure group had a higher mean preoperative AHI than patients who received other adjuvant procedures, which in context of the findings from the linear regression models, could partly explain the larger reduction in AHI observed postoperatively. Additional studies with larger sample sizes are needed to further explore this relationship and to better understand the potential benefits of adjuvant procedures. Patients who underwent adjuvant genioglossus advancement had either negligible or positive changes in AHI. There is limited data in literature on outcomes associated with genioglossus advancement, and it has largely fallen out of favor in the era of neurostimulation. Nevertheless, our data highlight the need for more research on hyoid suspension with adjuvant procedures targeting the tongue base.

Our results suggest that hyoid suspension’s effectiveness in alleviating OSA-associated symptoms is less robust compared to traditional first-line treatments such as weight loss and positive airway pressure (PAP) usage [24–27]. Additionally, hyoid suspension appears to be less effective than other surgical approaches, such as hypoglossal nerve stimulation [28–30]. However, for some patients with PAP intolerance and refractory OSA unresponsive to first-line therapies, hyoid suspension may still have a valuable role. Patients who wish to avoid device implantation and exhibit predominantly hypopharyngeal collapse on drug-induced sleep endoscopy may be good candidates for hyoid suspension. Furthermore, those with significant hypopharyngeal or base of tongue collapse and a high preoperative AHI who have exhausted other surgical options may benefit from this procedure. Although our analysis did not reveal any significant associations between DISE pattern of collapse and odds of surgical success, any meaningful conclusions are precluded by the small sample of patient with preoperative DISE (*n* = 16). Additional research is needed to determine optimal factors for patient selection.

Although the overall reduction in AHI and ESS in our study met statistical significance, this does not necessarily translate into clinically meaningful effects. The association of OSA with cardiovascular disease and metabolic syndrome is directly correlated with AHI, with a significantly lower risk among patients with mild OSA (AHI < 15 events/hour) [32]. In our study, it is notable that 44% of patients still had moderate to severe OSA post-operatively. In the analysis stratified by adjuvant procedure, only patients with hyoid suspension alone or adjuvant palatal surgery had mean reductions in AHI below the 15 events/hour threshold. Based on these findings, coupled with the surgical success rate of approximately 30%, hyoid suspension as part of multilevel OSA surgery likely only has a clinically meaningful impact for select patients.

This study is limited by its retrospective nature and single-institution scope. Given that patients in our sample had a relatively lower AHI compared to other studies, our findings may not be generalizable to all potential candidates for hyoid suspension surgery. Based on these differences in baseline characteristics, the overall mean change in AHI in our study is likely an underestimation. Additionally, most patients in our sample had adjuvant OSA surgeries at the time of hyoid suspension, so we are unable to estimate the effects of hyoid suspension alone. Further, given the small number of cases included in this review, we were unable to robustly analyze the effect of hyoid-mandibulopexy on OSA as separate from hyothyroidopexy. This was exacerbated by the confounding effect of adjuvant palatal procedures in 2 of the 5 total hyoid-mandibulopexy procedures. Additional limitations include potential unmeasured confounding factors such as changes in practice patterns and surgeon experience throughout the study period. Furthermore, given the small sample size, we had limited statistical power to detect true differences. We also did not have sleep architecture data beyond the polysomnogram outcomes reported in this study, which would be valuable context for interpreting the overall effects of surgery on sleep. Finally, given the retrospective nature of this study, we were not able to ascertain postoperative adverse events or complications for our patient cohort. Despite these limitations, our study adds to the evidence base for hyoid suspension as a viable treatment option for OSA, especially when combined with adjuvant palatal surgery.

## Conclusion

Our study demonstrates that hyoid suspension as part of multilevel OSA surgery leads to a statistically significant reduction in AHI and improvement in ESS in the treatment of OSA. Although the reductions in AHI and ESS were statistically significant, they may not translate to clinically meaningful outcomes for all patients Further research is needed to clarify the role of adjuvant procedures and factors to optimize patient selection.

## Electronic supplementary material

Below is the link to the electronic supplementary material.

Supplementary Material 1
