# Supplementary material for: Evaluating outcomes of hyoid suspension combined with other upper airway surgery for obstructive sleep apnea
Source: Sleep Breath. 2025 Jul 7;29(4):234. doi: 10.1007/s11325-025-03370-8 (PMC12234598; doi:10.1007/s11325-025-03370-8)
Supplement: Supplementary file 1 — Supplementary Material 1 [file 11325_2025_3370_MOESM1_ESM.docx]

**Supplementary Tables**

**Supplemental Table 1: DISE-specific analysis (available for 16 patients with pre-operative DISE data)**

|  | Number (frequency) | **Odds ratio and 95% CI for success based on Sher20 criteria** | **p-value** |
| --- | --- | --- | --- |
| **Velum** |  |  |  |
| No collapse | 2 (12.5%) | Reference |  |
| Partial anterior-posterior | 3 (18.8%) | 0.50 (0.01 to 19.6) | 0.711 |
| Complete anterior-posterior | 5 (31.3%) | 0.67 (0.03 to 18.1) | 0.810 |
| Partial concentric* | 2 (12.5%) | n/a | n/a |
| Complete Concentric | 4 (25.0%) | 0.33 (0.01 to 11.9) | 0.547 |
| **Oropharynx** |  |  |  |
| No collapse | 11 (68.8%) | Reference |  |
| Partial lateral wall | 5 (31.3%) | 6.75 (0.64 to 71.2) | 0.112 |
| Complete lateral wall* | 0 (0%) | n/a | n/a |
| **Tongue base** |  |  |  |
| No collapse | 4 (25.0%) | Reference |  |
| Partial anterior-posterior | 4 (25.0%) | 1.0 (0.04 to 24.5) | 1.000 |
| Complete anterior-posterior | 8 (50.0%) | 1.80 (0.12 to 26.2) | 0.667 |
| **Epiglottis** |  |  |  |
| No collapse | 8 (50.0%) | Reference |  |
| Partial anterior-posterior | 4 (25.0%) | 1.00 (0.06 to 16.0) | 1.000 |
| Complete-anterior posterior | 4 (25.0%) | 3.00 (0.24 to 37.7) | 0.395 |

*We were unable to estimate odds ratios for velum partial concentric collapse and oropharynx complete lateral wall collapse due to small sample size

**Supplemental Table 2: Outcomes in patients who underwent an adjuvant nasal procedure (n=16)**

| Pre-operative AHI (mean, SD) | 33.5 (23.7) |
| --- | --- |
| Change in AHI (mean, SD) | -5.4 (27.6) |
| Change in O2 Nadir (mean, SD) | 4.8 (12.9) |
| Change in ESS (mean, SD) | -0.67 (3.7) |
| Success based on Sher20 criteria | 4 (25.0%) |

**Supplemental Table 3: Stratified analysis of OSA outcomes among patients who underwent hyothyroidopexy (n=38)**

|  | **Pre-operative (mean, SD)** | **Post-operative (mean, SD)** | **Mean difference (95% CI)** | **p-value** |
| --- | --- | --- | --- | --- |
| AHI | 30.6 (21.9) | 22.6 (24.5) | -7.9 (-16.6 to 0.60) | 0.068 |
| O2 Nadir | 82.9 (13.3) | 85.4 (7.2) | 2.4 (-0.89 to 5.62) | 0.148 |
| ESS | 8.3 (4.8) | 6.3 (5.6) | -1.0 (-2.9 to 0.85) | 0.265 |

**Supplemental Table 4: Stratified analysis of OSA outcomes among patients who underwent hyoid-mandibulopexy procedure (n=5)**

|  | **Pre-operative (mean, SD)** | **Post-operative (mean, SD)** | **Mean difference (95% CI)** | **p-value** |
| --- | --- | --- | --- | --- |
| AHI | 31.4 (15.9) | 11.2 (6.2) | -20.1 (-37.7 to -2.6) | 0.033 |
| O2 Nadir | 77.8 (9.1) | 86.3 (4.8) | 8.5 (-9.1 to 26.1) | 0.221 |
| ESS | 14.0 (4.3) | 8.5 (4.4) | -5.5 (-7.6 to -3.4) | 0.003 |
